# The course of diabetes in children, adolescents and young adults: does the autoimmunity status matter?

**DOI:** 10.1186/s12902-016-0145-3

**Published:** 2016-11-15

**Authors:** Rasa Verkauskiene, Evalda Danyte, Rimante Dobrovolskiene, Ingrida Stankute, Diana Simoniene, Dovile Razanskaite-Virbickiene, Audrone Seibokaite, Brone Urbonaite, Nijole Jurgeviciene, Astra Vitkauskiene, Valerie Schwitzgebel, Dalia Marciulionyte

**Affiliations:** 1 Institute of Endocrinology, Medical Academy, Lithuanian University of Health Sciences, Eiveniu 2, LT-50161 Kaunas, Lithuania; 2 Department of Endocrinology, Hospital of Lithuanian University of Health Sciences Kauno Klinikos, LT-50161 Kaunas, Lithuania; 3 Department of Child and Adolescent, University Hospital of Geneva, 1211 Geneva, Switzerland; 4 Diabetes Center, Faculty of Medicine, University of Geneva, 1211 Geneva, Switzerland

**Keywords:** Type 1 diabetes, Children, Adolescents, Young adults, Pancreatic antibodies

## Abstract

**Background:**

Initial classification of diabetes of young may require revision to improve diagnostic accuracy of different forms of diabetes.

The aim of our study was to examine markers of beta-cell autoimmunity in a cohort of young (0–25 years) patients with type 1 diabetes and compare the presentation and course of the disease according to the presence of pancreatic antibodies.

**Methods:**

Cross-sectional population-based study was performed covering 100% of pediatric (*n* = 860) and 70% of 18–25 years old adult patients (*n* = 349) with type 1 diabetes in Lithuania.

**Results:**

No antibodies (GAD65, IA-2, IAA and ICA) were found in 87 (7.5%) cases. Familial history of diabetes was more frequent in those with antibodies-negative diabetes (24.1 vs. 9.4%, *p* < 0.001). Gestational age, birth weight and age at diagnosis was similar in both groups. Ketosis at presentation was more frequent in patients with autoimmune diabetes (88.1 vs. 73.5%, *p* < 0.05). HbA1c at the moment of investigation was 8.6 (3) vs. 8.7 (2.2)% in antibodies-negative and antibodies-positive diabetes groups, respectively, *p* > 0.05. In the whole cohort, neuropathy was found in 8.8% and nephropathy - in 8.1% of cases, not depending on autoimmunity status. Adjusted for age at onset, disease duration and HbA1c, retinopathy was more frequent in antibodies-negative subjects (13.8 vs. 7.8%, *p* < 0.05).

**Conclusion:**

Antibodies-negative pediatric and young adult patients with type 1 diabetes in this study had higher incidence of family history of diabetes, higher frequency of retinopathy, less frequent ketosis at presentation, but similar age at onset, HbA1c, incidence of nephropathy and neuropathy compared to antibodies-positive patients.

## Background

Diabetes at young age is predominantly type 1 diabetes - a chronic autoimmune disease, caused by an interplay of genetic susceptibility and environmental factors [1, 2]. The incidence of type 1 diabetes is rising worldwide, particularly in young children [1, 3, 4], and varies from the lowest values reported in China and Venezuela (0.1 per 100,000 inhabitants per year) to the highest recorded in Finland (42.9 per 100,000 inhabitants per year) [2–4]. DIAMOND project results showed the average 2.8% of annual increase in incidence of type 1 diabetes calculated from data of 103 centers [5]. The standard incidence of type 1 diabetes in Lithuania is 10.42 (95℅ - CI 10.01–10.85) per 100,000 persons aged 0–14 years and the average of annual incidence increase by 4.75℅ (Urbonaite B, unpublished data).

The risk for developing type 1 diabetes increases with genetic susceptibility and the presence of immunological markers of beta-cell autoimmunity. Antibodies may be used as markers of beta-cell destruction and reflect disease severity [6, 7]. Type 1 diabetes is characterized by the presence of antibodies to a 65 kD glutamic acid decarboxylase antigen (GAD65), antibodies against protein tyrosine phosphatase (IA-2), insulin antibodies (IAAs), islet cell antibodies (ICAs) and zinc transporter 8 (ZnT8A) in blood that identify the autoimmune process leading to beta-cell destruction [8]. A recent experience shows that the initial classification of diabetes of young requires revision in about 10% of cases in whom monogenic forms of diabetes might imply different treatment modalities [9]. Therefore, an in depth characterization of young patients with diabetes, including the analysis of pancreatic autoimmune antibodies is mandatory to improve diagnostic accuracy of the different forms of diabetes.

The aim of our study was to examine markers of beta-cell autoimmunity in a cohort of young (0-25 years of age) patients with type 1 diabetes and compare the presentation and course of the disease according the presence of immunological markers.

## Methods

### Study design and subjects

A cross sectional population-based study was conducted in a single research center as a part of joint Lithuanian–Swiss project “Genetic Diabetes in Lithuania”. The principal aim of this project is to screen all patients with type 1 diabetes under the age of 25 years in Lithuania for autoimmune antibodies in order to select patients for genetic testing for monogenic diabetes and to compare clinical presentation of antibodies-positive and antibodies-negative subjects.

Our cohort consisted of 1209 subjects covering all pediatric patients (<18 years, *n* = 860), and part of adult patients younger than 25 years (*n* = 349) diagnosed with type 1 diabetes. All patients had a physician diagnosis of type 1 diabetes between March 1990 and March 2015. According to the national Lithuanian type 1 diabetes registry, the study cohort covered 100% of all pediatric and 70% of 18–25 years old adult patients with type 1 diabetes in Lithuania.

Patients with diagnosed type 1 diabetes (up to 25 years of age, regardless of the duration of the disease) were identified from Lithuanian national type 1 diabetes database and invited to participate in the study on their visit to the family doctor and/or endocrinologist, a pediatric endocrinologist, meetings of diabetes societies, as well as by post, e-mails and phone calls. Patients were recruited and enrolled into the study between January 2013 and April 2015. 164 patients were newly diagnosed with type 1 diabetes at study entry, and in 1045 subjects diabetes duration was from 2 weeks to 24.7 years.

At the study entry, data on age at onset of diabetes, diabetes duration, gestational age and birth weight, the daily insulin dose, presentation of the disease and family history of diabetes were collected. Clinical examination included height (cm), weight (kg), arterial blood pressure, heart rate, examination of feet and eye fundus. The data on gestational age and birth weight were selected from medical documentation when available and from questionnaire.

At study entry, all study subjects were investigated for pancreatic autoimmune markers (GAD65, IA-2, IAAs, ICAs) and coexistence of other autoimmune diseases (thyroid, celiac disease). Diabetes control was assessed by levels of HbA1c. Fasting blood samples were taken for analysis of lipid profile. Patients were all screened for diabetes complications: retinopathy, nephropathy and neuropathy.

### Laboratory analyses

Glycosylated hemoglobin (HbA1c), lipids, serum creatinine were measured by UniCel DxC 800 Synchron system (Beckman Coulter, USA). The normal cut-off values of HbA1c were 4–6% (20 mmol/mol – 42 mmol/mol). Optimal metabolic control was defined by HbA1c <7.5% (58 mmol/mol) for children [10] and adults [11]. Dyslipidemia was defined as LDL-cholesterol ≥2.59 mmol/l, HDL-cholesterol ≤1.55 mmol/l, total cholesterol ≥5.2 mmol/l for patients ≥16 years and ≥5.5 for children under 16 years, and triglycerides ≥1.95 mmol/l. Dyslipidemia was considered present if one or more of these lipid or lipoprotein levels were abnormal.

Ketosis at diagnosis was defined as presence of ketonuria (urine ketone body 1+ and above) or diabetic ketoacidosis [10].

#### Thyroid antibodies (ATPO) and thyroid hormones (TSH, FT4)

ATPO (normal range, 0–78 U/ml), TSH (normal range 0.27–3.5 mU/ml) and FT4 (normal range 10–22 pmol/l) were measured using open analyzer system on a radioactive basis for radioimmunoassays SR300 (Stratec Biomedical Systems AG (Germany)).

Thyroid function was evaluated as hypothyroidism, subclinical hypothyroidism, hyperthyroidism and subclinical hyperthyroidism. Subclinical hypothyroidism was diagnosed when FT4 levels were within normal reference laboratory range but TSH levels were elevated. Subclinical hyperthyroidism was defined as TSH level below 0.27 mU/ml and FT4 within normal range.

*Tissue transglutaminase IgA antibodies (tTG-A)* were measured by enzyme immunoassay (ELISA) kits, using Gemini analyzer (Stratec Biomedical Systems AG (Germany)). Concentrations >18 U/ml for tTG-A were considered positive, - between 12 and 18 U/ml – borderline and concentrations <12 U/ml - normal.

#### Pancreatic antibodies

GAD65, IA-2, IAAs antibodies were measured by radioimmunoassay (RIA) using γ-ray counter BERTHOLD (Germany), and ICAs antibodies were measured by enzyme immunoassay (ELISA) and evaluated by means of microtiter plate reader STAT FAX2100 (USA). DEMEDITEC’s Diagnostics GmbH (Germany) assay kits were used, as previously described [12–14].

GAD65 antibodies measuring range was 1–300 U/ml. The lowest detection limit at +2SD was 0.11 U/ml. Assay’s negative cut-off was ≤1.0 U/ml, and positive >1.0 U/ml. Inter-assay coefficient of variation (CV) was 6.9%, intra-assay CV-3.7%, specificity and sensitivity were 95% and 84%, respectively.

IA-2 antibodies RIA assay measuring range was 1–50 U/ml. The lowest detection limit at +2 SD was 0.16 U/ml. Assay’s negative cut-off was ≤1.0 U/ml and positive - > 1.0 U/ml. Inter-assay CV was 5.3%, intra-assay CV - 2.8%, specificity and sensitivity were 100% and 70%, respectively.

IAAs antibodies measuring range was 0.4–50 U/ml. The lowest detection limit at +2 SD was 0.03 U/ml. Assay’s negative cut-off was < 0.4 U/ml, and positive - ≥0.4 U/ml. Inter-assay CV was 8.0%, and intra-assay CV - 3.3%.

ICAs antibodies assay is qualitative ELISA test for in vitro detection of circulating IgG antibodies against islet cell antigens in human serum [14]. Samples with optical density ratio values ≤0.95 show a low level of ICAs antibodies (negative result), values >0.95 show a high level (positive result).

### Evaluation of microvascular diabetes complications

#### Retinopathy

Retina examination was performed by a single diabetes ophthalmologist. The digital fundus photographies were used for the evaluation of diabetic eye disease.

#### Albumin excretion rate (AER)

24 hour urine albumin excretion rate (AER) was calculated as described previously [15] and defined as normal when AER < 30mg/24h; microalbuminuria–when AER 30-300 mg/24h, macroalbuminuria–when AER > 300 mg/24h.

#### Neuropathy

Clinical neuropathy was defined as the presence of symptoms and signs consistent with distal symmetrical peripheral neuropathy. Michigan Neuropathy Screening Questionnaire was applied and vibration sensation was tested in the great toe using a 128-Hz tuning fork, pressure sensation test with Semmes-Weinstein 10g monofilament and temperature sensation test with thermal sensitivity tester Tip Therm were used for neuropathy screening. Peripheral neuropathy was diagnosed when two or more of the tests were abnormal [16, 17].

### Statistical analyses

Statistical analyses were performed using SPSS software version 20.0. The data were evaluated using Student’s 2-tailed *t* test, *χ* ^2^ statistics, parametric one way ANOVA (in the case of normal distribution) and Mann–Whitney *U*-test (in the case of non-normal distribution) or Kruskall Wallis one-way ANOVA (in the case of ordinal data). *P* values <0.05 were assigned statistical significance. All *P* values are 2-tailed.

## Results

### General characteristics of the cohort

**Fig. 1:**
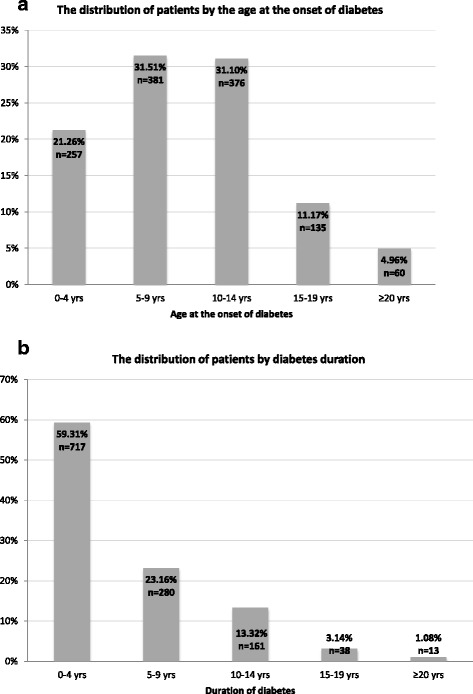
The distribution of patients by age at the onset of diabetes (**a**) and diabetes duration (**b**) groups

The mean age at the onset of diabetes was 9.9 (5.3) years (0.01–24.8 years, median 9.7 years). In 4 cases the age at onset of diabetes was less than 6 months, corresponding to neonatal diabetes form, confirmed later with genetic testing and identification of mutation in *KCNJ11* gene. The peaks of onset of diabetes occurred in two age groups: 5–9 years and 10–14 years (Fig. 1). The mean age of patients was 15 (6.2) years. The mean duration of diabetes was 5.1 (5) years (0.01–24.7, median 3.8 years). No gender predominance was apparent in our cohort (males 48.5%).

### Autoimmunity status

**Fig. 2:**
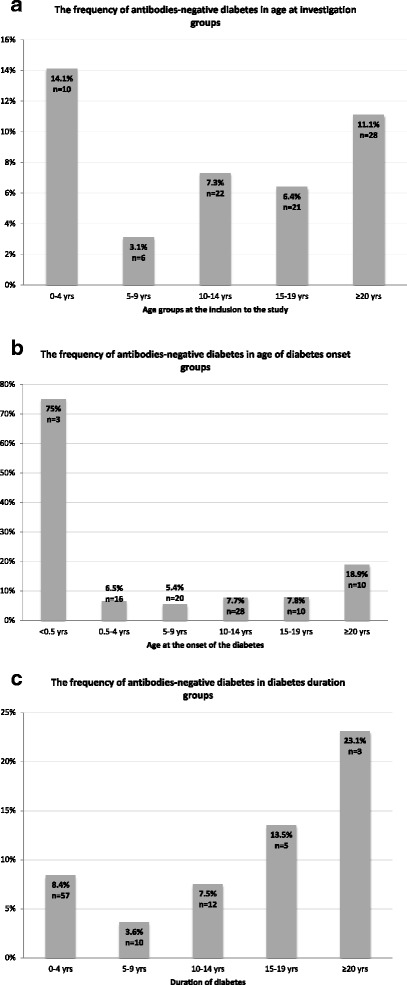
The frequency of antibodies-negative diabetes in age at investigation (**a**), age at the onset of diabetes (**b**) and diabetes duration groups (**c**)

No immunological markers of beta-cell autoimmunity were found in 87 cases (7.5%) (Table 1) of the whole cohort, and in 20 cases (12.2%) among newly diagnosed diabetic patients (Table 3). Four patients with neonatal diabetes (onset before 6 months of age) were on insulin treatment at the time of investigation; in 3 cases no antibodies were found, and IAAs were present in one case. All negative immunological markers were found more frequently in the youngest (0–4 years) and the oldest (20–24 years) patients groups, and with the duration of diabetes ≥14 years (Fig. 2). Positive ICAs were observed least frequently in the whole cohort (Table 2) and in newly diagnosed diabetic patients (Table 3).

**Table 1:** Frequency of various antibody combinations in patients with diabetes

	Number	Frequency (%)
No detectable antibodies	87	7.5
Single Ab +		
GAD65 +	38	3.3
IA-2 +	50	4.3
IAAs +	178	15.3
ICAs +	8	0.7
Two Ab’s +		
GAD65 + and IA-2 +	415	35.6
GAD65 + and IAAs +	458	39.3
GAD65 + and ICAs +	38	3.3
IA-2 + and IAAs +	505	43.3
IA-2 + and ICAs +	42	3.6
IAAs + and ICAs +	64	5.5
Three Ab’s +		
GAD65 +, IA-2 +, IAAs +	298	25.6
GAD65 +, IA-2 +, ICAs +	25	2.1
GAD65 +, IAAs +, ICAs +	31	2.7
IA-2 +, IAAs+, ICAs +	34	2.9
All four Ab’s +	30	2.6

*Ab* antibody, *GAD65 *antibodies against glutamic acid decarboxylase antigen 65 kD, *IA-2* antibodies against protein tyrosine phosphatase, *IAAs* insulin antibodies, *ICAs* islet cell antibodies

**Table 2:** Comparison of clinical features between groups of DM patients according to autoimmunity status

	No Ab’s (*n* = 87; 7.5%)	≥1 Ab + (*n* = 1079; 92.5%)	Only GAD65 + (*n* = 38; 3.3%)	Only IA-2 + (*n* = 50; 4.3%)	Only ICAs + (*n* = 8; 0.7%)	Only IAAs + (*n* = 178; 15.3%)	2 Ab’s + (*n* = 477; 40.9%)	3 Ab’s + (*n* = 308; 26.4%)
Age (yrs)	15.87	15 (6)	16.5 (7)	14 (7.3)	17.8 (4.4)	15.7 (5.7)	14.9 (6.1)	15 (5.9)
Age at onset of DM (yrs)	10.6 (6.3)	9.8 (5.2)	12 (6.9)	9.3 (5.5)	10.9 (4.4)	7.9 (5.7)	9.8 (5.2)	9.6 (5.4)
Duration of DM (yrs)	5.3 (6.3)	5.1 (4.8)	4.4 (6)	4.7 (5.8)	5.9 (4.9)	5.4 (5)	5.2 (4.9)	5.4 (5)
Birth weight (gr)	3473 (568)	3515 (502)	3389 (389)	3619 (497)	3638 (680)	3492 (507)	3492 (507)	3501 (488)
Gestational age (weeks)	39.6 (1.2)	39.4 (1.6)	39.4 (1.2)	39.3 (2)	39.5 (1.2)	39.6 (1.2)	39.5 (1.4)	39.4 (1.6)
Males (%)	48.3	48.5	39.5	58	75	46.6	48.4	51
Positive family history of diabetes (1^st^ degree relatives) (%)	24.1	9.4^b^	2.6^c^	6^d^	37.5	10.1	10.5	11
HbA1c (%)	8.6 (3)	8.7 (2.2)	10 (2.5)^e^	9.5 (2.1)	7.9 (2.3)	8.6 (1.9)	8.6 (2.1)	8.6 (2.1)
Insulin dose (U/kg/d)	0.63 (0.4)	0.77 (0.4)^f^	0.81 (0.4)^g^	0.74 (0.3)^h^	0.81(0.4)	0.79 (0.4)^i^	0.76 (0.4)	0.74 (0.4)
Positive ATPO (%)	3.1	14.6^j^	11.	6.7	16.	11.6	13.6	15.7
Positive tTG-A (%)	0	3.9	9.1	2.6	0	3.9	2.9	3.9
Dyslipidemia (%)	83.9	85.7	89.5	90	87.5	86	83.6	85.7
Ketosis (data available for 621 patients)	73.5% (*n* = 25/34^a^)	88.1% (*n* = 517/587)^k^	84% (*n* = 21/25^a^)	100% (*n* = 21/21^a^)^l^	100% (*n* = 2/2^a)^	87.6 (*n* = 92/157^a^)^m^	86% (*n* = 227/264^a^)^n^	92.4% (*n* = 145/157^a^)^o^
Retinopathy	13.8% (*n* = 12)	7.8% (*n* = 84)^p^	7.9% (*n* = 3)	12% (*n* = 6)	25% (*n* = 2)	9.1% (*n* = 28)	8.4% (*n* = 40)	9.1% (*n* = 28)
Neuropathy	10.8% (*n* = 9)	8.7% (*n* = 91)	5.7% (*n* = 2)	14% (*n* = 7)	0	8.6% (*n* = 26)	10.2% (*n* = 47)	8.6% (*n* = 26)
Nephropathy (AER > 300 mg)	4.8% (*n* = 4)	8.8% (*n* = 91)	7.9% (*n* = 3)	2% (*n* = 1)	12.5% (*n* = 1)	9.2% (*n* = 27)	8% (*n* = 37)	9.2% (*n* = 27)

All parameters are presented as mean (±SD) values unless otherwise stated

*Ab* antibody, *GAD65 *antibodies against glutamic acid decarboxylase antigen 65 kD, *IA-2* antibodies against protein tyrosine phosphatase, *IAAs* insulin antibodies, *ICAs* islet cell antibodies, *DM* diabetes mellitus, *yrs* years, *gr* grams, *HbA1c* glycosylated hemoglobin, *ATPO *thyroid peroxidase antibodies, *tTG-A* tissue transglutaminase antibodies, *AER* albumin excretion rate

^a^ - present ketosis out of whom data was available

*P* values compared with the group of all negative antibodies: ^b^ 0.000015, ^c^ 0.004, ^d^ 0.009, ^e^ 0.014, ^f^ 0.001, ^g^ 0.034, ^h^ 0.004, ^i^ 0.001, ^j^ 0.01, ^k^ 0.002, ^l^ 0.009, ^m^ 0.011, ^n^ 0.033, ^o^ 0.011, ^p^ 0.046

**Table 3:** Comparison of clinical features between groups of newly diagnosed DM patients (*n* = 164) according to autoimmunity status

	No Ab’s (*n* = 20; 12.2%)	≥1 Ab + (*n* = 144; 87.8%)	Only GAD65 + (*n* = 11; 6.7%)	Only IA-2 + (*n* = 19; 11.6%)	Only ICAs + (*n* = 2; 1.2%)	Only IAAs + (*n* = 3; 1.8%)	2 Ab’s + (*n* = 78; 47.6%)	3 Ab’s + (*n* = 28; 17.1%)
Age at onset of DM (yrs)	15.8 (6.5)	11.5 (6.1) ^b^	16.1 (7.3)	9.8 (6) ^f^	14.7 (1.2)	10.9 (8.7)	12.1 (5.5) ^h^	8.5 (5.8)^k^
Birth weight (gr)	3643 (699)	3470 (485)	3312 (421)	3628 (443)	3200	2920 (1018)	3477 (502)	3475 (408)
Gestational age (weeks)	39.4 (1)	39.6 (1.5)	39.2 (1.8)	39.6 (1.3)	40	40	39.6 (1.7)	39.7 (1)
Males (%)	45	45.1	18.2	57.9	50	33.3	46.2	50
Positive family history of diabetes (1^st^ degree relatives) (%)	10	11.1	9.1	10.5	50	33.3	10.3	10.7
HbA1c (%)	9.9 (3.7)	10.9 (2.3)	11.5 (2.2)	10.8 (1.5)	6.2 (0.1)	7.7 (1.4)	11.3 (2.3)^i^	10.3 (2.3)
Insulin dose (U/kg/d)	0.43 (0.3)	0.71 (0.4) ^c^	0.8 (0.4) ^e^	0.77 (0.3) ^g^	0	0.39 (0.3)	0.75 (0.4) ^j^	0.62 (0.4)
Positive ATPO (%)	5.9	14.2	28.6	0	0	0	18.3	8.3
Positive tTG-A (%)	0	7.4	20	6.3	100	--	8.2	5
Dyslipidemia (%)	80	86.8	81.8	94.7	100	100	84.6	85.7
Ketosis (data available for 37 patients)	50%(*n* = 4/8^a^)	13.8% ^d^(*n* = 4/29^a^)	14.3%(*n* = 1/7^a^)	100%(*n* = 3/3^a^)	--	--	86.7% (*n* = 13/15^a^)	100% (*n* = 3/3^a^)

All parameters are presented as mean (±SD) values unless otherwise stated

*Ab* antibody, *GAD65 *antibodies against glutamic acid decarboxylase antigen 65 kD, *IA-2* antibodies against protein tyrosine phosphatase, *IAAs* insulin antibodies, *ICAs* islet cell antibodies, *DM* diabetes mellitus, *yrs* years, *gr* grams, *HbA1c* glycosylated hemoglobin, *ATPO *thyroid peroxidase antibodies, *tTG-A* tissue transglutaminase antibodies

^a^ - present ketosis out of whom data was available

*P* values compared with the group of all negative antibodies: ^b^0.004, ^c^ 0.002, ^d^ 0.049, ^e^ 0.008,^f^ 0.005, ^g^ 0.001, ^h^ 0.012, ^i^ 0.042, ^j^ 0.001,^k^ 0.0002

**Fig. 3:**
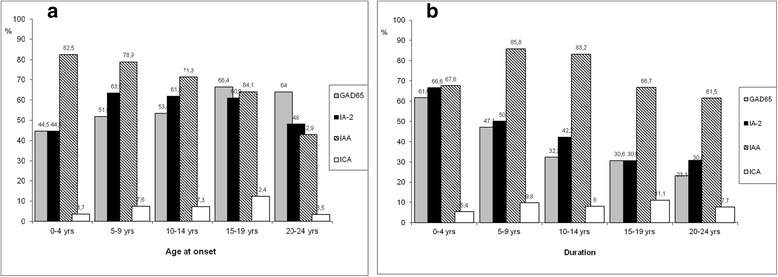
**a** Frequency of positive antibodies among age the onset of diabetes groups. **b** Frequency of positive antibodies according to diabetes duration

**Fig. 4:**
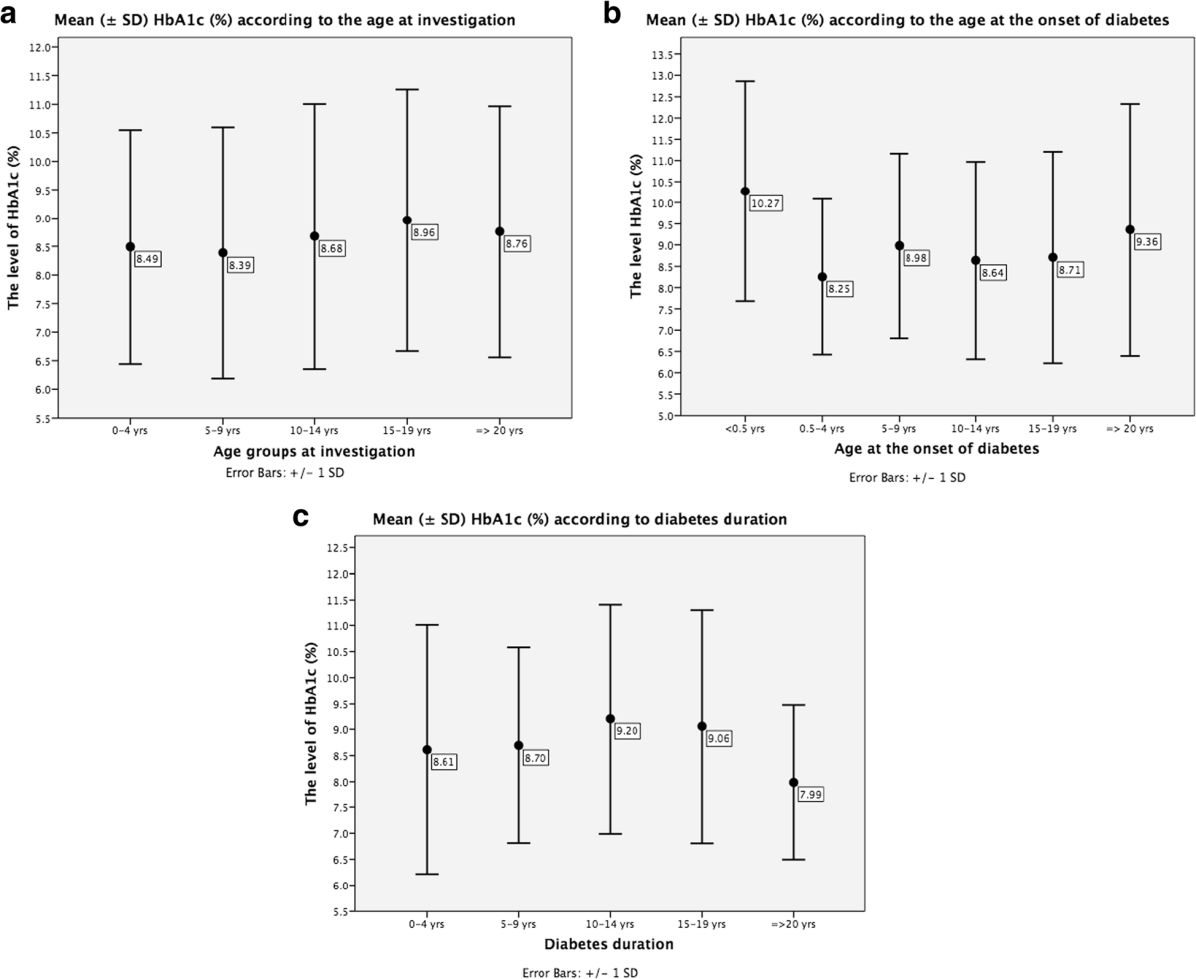
Mean (± SD) HbA1c (%) according to: (**a**) age at investigation (*p* = 0.004 between age groups 5–9 and 15–19 years); (**b**) age at onset of diabetes (*p* < 0.05 between age at onset 0.5–4 years group and 5–9, 10–14, 15–19 and ≥20 year groups; *p* = 0.036 between age at onset groups 5–9 and 10–14 years); (**c**) diabetes duration (*p* = 0.003 between groups 0–4 and 10–14 years; *p* = 0.024 between groups 5–9 and 10–14 years)

Positive IA-2 antibodies were found in two thirds of patients with the age at onset of diabetes 5–19 years, whereas higher frequency of GAD65 antibodies was observed in older ones and was found in two thirds with age at onset 15–24 years (Fig. 3). The frequency of both positive GAD65 and IA-2 was higher in patients with longer duration of the disease (Fig. 4).

### Birth data and family history of diabetes

The mean gestational age in our cohort was 39.4 (1.6) weeks, 4.1% were born preterm (<37 gestational weeks). 0.1% of subjects were born of very low birth weight (<1500 g) and 2.2% were born of low birth weight (<2500 g). There were no differences neither in birth data nor gender distribution according to autoimmunity status (Table 2).

Almost one third (31.9%) of patients had positive family history of diabetes among first, second or third-degree relatives. The first degree relatives had diabetes in 10.8% of study cases, significantly more frequently in antibodies-negative subjects (Table 2). Second degree relatives with diabetes were reported in 26.2%, and third degree relatives- in 16.4% of cases. 8 (0.7%) patients had very strong positive family history among first, second and third-degree relatives.

### Disease presentation

Age at onset of diabetes was similar in antibodies-negative and antibodies-positive (with at least one positive antibody) patients. However, patients with 3 and more positive antibodies were significantly younger at disease onset compared to antibodies-negative subjects (Table 2).

Ketosis at the time of diagnosis was present in 542 (87.3%) of 621 cases in whom data were available. Ketosis was significantly more frequent in patients with no family history of diabetes (91.2% vs. 78.3%, *p* < 0.05) and in the antibodies-negative group (88.1 vs. 73.5%, *p* < 0.05). In the subcohort with newly diagnosed diabetes, ketosis was also more frequent among those with all negative autoimmunity markers than with at least one positive antibody (Table 3).

### Diabetes management and control

**Fig. 5:**
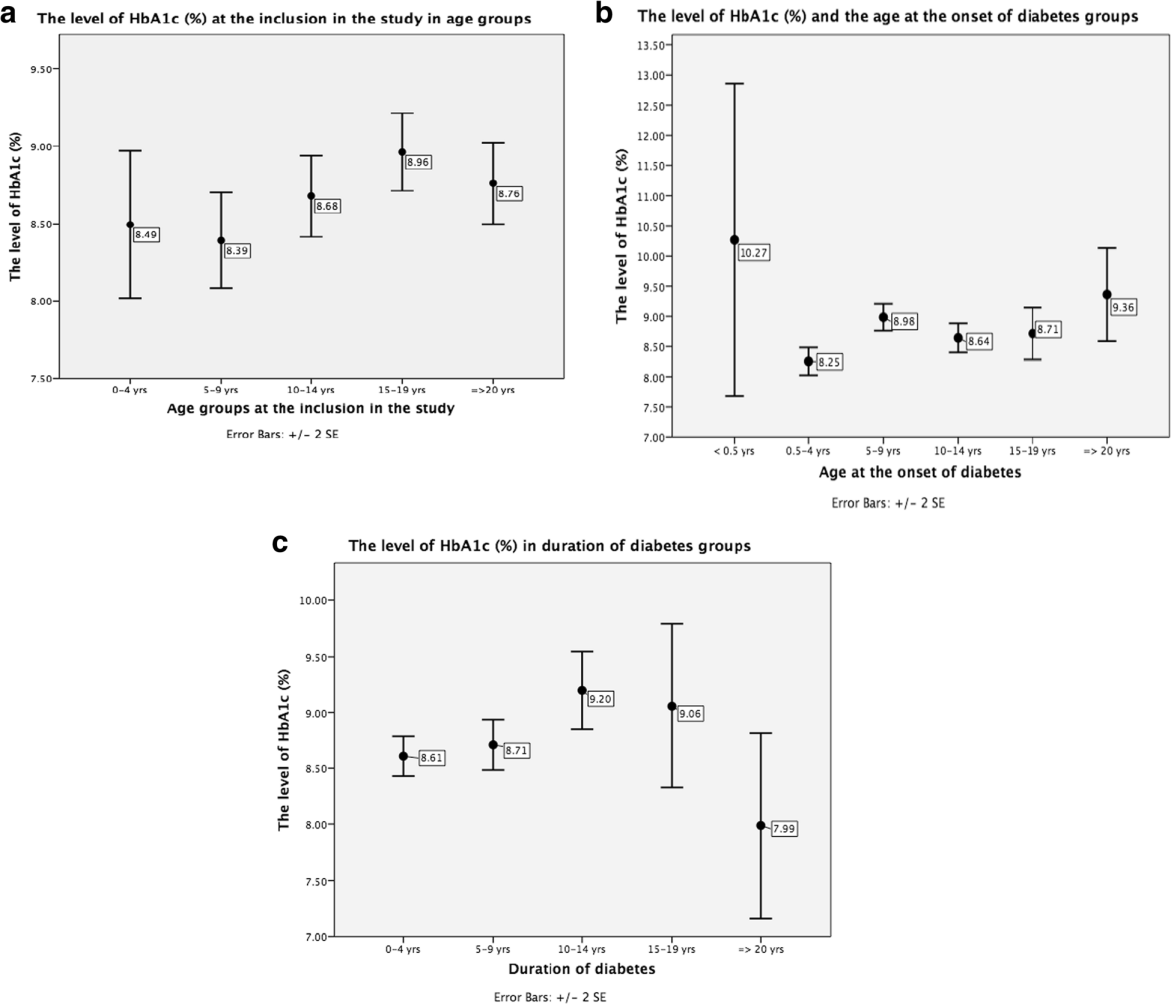
Mean (± SE) HbA1c (%) according to the age at investigation (**a**), age at the onset of diabetes (**b**) and diabetes duration (**c**)

In the whole cohort, mean HbA1c at the study entry was 8.7 (2.3)% (72 (2) mmol/mol) (4.7–19.9% (28–194 mmol/mol), median 8.3% (67 mmol/mol)). The highest HbA1c was observed in adolescents and young adults groups (Fig. 5), although neither age group had optimal glycemic control. Males had slightly but significantly better glycemic control than females (8.5 (2.1)% (69 (1) mmol/mol) vs. 9 (2.3)% (74 (2) mmol/mol), *p* < 0.05). HbA1c was similar between patients in antibodies-negative and antibodies-positive subjects; however, comparisons of patients with different antibodies types to antibodies-negative ones revealed significantly higher HbA1c in those positive for only GAD65 antibodies (Table 2).

The mean daily insulin dose in the whole cohort was 0.79 (0.35) U/kg/d. The mean insulin dose in antibodies-negative patients was significantly lower than in patients with at least one positive antibody in the whole cohort (0.63 (0.4) vs. 0.77 (0.4), *p* = 0.001, respectively) (Table 2), and also in the subcohort with newly diagnosed T1D patients (0.43 (0.3) vs. 0.71 (0.4), *p* = 0.002, respectively) (Table 3).

### Microvascular complications and dyslipidemia

Neuropathy was present in 100 (8.8%) and retinopathy - in 96 (8.2%) of our patients. There was one case of diabetic cataract in a 14 years old adolescent. Elevated AER was found in 94 (8.1%) cases. Adjusted for age at onset, disease duration and HbA1c, antibodies-negative patients had significantly higher incidence of retinopathy, compared with antibodies-positive subjects, and similar incidence of neuropathy and nephropathy, although the latter was almost two times less frequent in antibodies-negative group, but the difference did not reach significance, possibly due to small numbers of patients with nephropathy in the antibodies-negative group (Table 2). We found significant relationships between age, duration of diabetes, glycemic control, insulin dose and all microvascular complications. Detailed comparison of patients with and without microvascular complications is shown in Table 4.

**Table 4:** Clinical characteristics and autoimmunity status according to presence of diabetic microvascular complications

	Retinopathy		Neuropathy		AER		No microvascular alterations
	Present	Absent	Present	Absent	Elevated	Normal	
Age at the onset (yrs)	8.1 (4.3)^b^	10 (5.4)	9.9 (5)^h^	9.7 (5.2)	9.9 (5.1)^n^	9.8 (5.3)	9.9(5.4)
	*p* = 0.001		*p* = 0.655		*p* = 0.821		
Age at study entry (yrs)	20.7 (3.8)^c^	14.5 (6)	20.6 (4.2)^i^	14.2 (5.9)	(4.2)^o^	14.2 (5.9)	14 (6)
	*p* = 0.0001		*p* = 0.0001		*p* = 0.0001		
Duration (yrs)	12.6 (4.8)^d^	4.5 (4.4)	10.7 (5)^j^	4.5 (4.5)	10.7 (5)^p^	4.5 (4.5)	4.1 (4.2)
	*p* = 0.0001		*p* = 0.0001		*p* = 0.0001		
HbA1c (%)	10 (2.3)^e^	8.6 (2.2)	9.3 (2.3)^k^	8.7 (2.2)	9.3 (2.3)^q^	8.7 (2.2)	8.5 (2.2)
	*p* = 0.0001		*p* = 0.005		*p* = 0.001		
Insulin dose (U/kg/d)	0.91 (0.3)^f^	0.78 (0.3)	0.86 (0.3)^l^	0.78 (0.3)	0.86 (0.3)^r^	0.78 (0.3)	0.74 (0.4)
	*p* = 0.0001		*p* = 0.01		*p* = 0.044		
All negative antibodies (%)^a^	12.5^g^	7	9^m^	7.2	9^s^	7.2	7.2
	*p* = 0.046		*p* = 0.637		*p* = 0.456		
GAD65 Ab levels (U/ml)	6.5 (18.6)	11.6 (28.9)	11.4 (29.5)	11.1 (28.2)	8.3 (18.9)	11.4 (28.9)	11.6 (29.3)
	*p* = 0.092		*p* = 0.913		*p* = 0.307		
IA-2 Ab levels (U/ml)	1.6 (3.3) ^t^	5.8 (10.7)	2.5 (5.7)^w^	5.7 (10.6)	5.4 (13.8)	5.5 (10)	5.9 (10.4)
	*p* = 0.0001		*p* = 0.003		*p* = 0.975		
IAAs levels (U/ml)	7.2 (10.2) ^x^	11.4 (15.3)	9 (15.7)	11.3 (14.9)	7.2 (11.1) ^y^	11.4 (15.2)	11.8 (15.3)
	*p* = 0.007		*p* = 0.145		*p* = 0.008		

All parameters are presented as mean (±SD) values unless otherwise stated

yrs years, HbA1c glycosylated hemoglobin, AER albumin excretion rate, *Ab* antibody, *GAD65* antibodies against glutamic acid decarboxylase antigen 65 kD, *IA-2* antibodies against protein tyrosine phosphatase, *IAAs* insulin antibodies

^a^-comparisons between groups adjusted for age at the onset of diabetes, duration and HbA1c

*P* values comparing the group with microvascular complication with the groups without microvascular alterations: ^b^ 0.002, ^c^ 0.0001, ^d^ 0.0001, ^e^ 0.0001, ^f^ 0.001, ^g^ 0.066, ^h^ 0.889, ^i^ 0.0001, ^j^ 0.0001, ^k^ 0.001, ^l^ 0.005, ^m^ 0.527, ^n^ 0.886, ^o^ 0.0001, ^p^ 0.0001, ^q^ 0.0001, ^r^ 0.032, ^s^ 0.394, ^t^ 0.001, ^w^ 0.006, ^x^ 0.004, ^y^ 0.003

84.9% (*n* = 990) of the cohort had abnormal lipid profile. Patients with dyslipidemia had higher HbA1c ((8.8 (2.3)% vs. 8.1 (1.8)%, *p* < 0.05), but also longer duration of DM (5.2 (5) vs. 5 (4.9) yrs; *p* = 0.572). Presence of pancreatic antibodies was not related to the incidence of dyslipidemia (Table 2).

### Co-existing autoimmune conditions

Thyroid antibodies (ATPO-Abs) were found in 13.8% of the entire cohort, significantly more frequently among patients with at least one positive pancreatic antibody (14.6% vs. 3.1%, respectively). Thyroid dysfunction was detected in 18.8% of cases: hypothyroidism was evident in 0.63%, subclinical hypothyroidism – in 16.9%, subclinical hyperthyroidism – in 1.26% of cases. Thyroid dysfunction was significantly more prevalent in females (*p* = 0.014).

Positive tTG-A were detected in 3.7% of patients, only in antibodies-positive subjects. Highest frequency of positive tTG-A was found in the youngest age at diagnosis group (7.4% in 0–4 years of age at diabetes onset group).

## Discussion

The main strength of this study is the clinical and immunological characterization of a national cohort of all children and the majority of young adults up to 25 years of age with type 1 diabetes.

Epidemiological data on childhood diabetes (0–14 years) in Lithuania are registered since 1983 [18, 19]. According to national registry, average annual type 1 diabetes incidence increase is 4.75%, and that of prevalence - 6.5%; therefore, the population of young people with diabetes constantly increases [20]. Traditionally, the diagnosis of diabetes is based on clinical data only, the age at onset of hyperglycemia without obesity being the main diagnostic criteria.

The appearance of autoantibodies to one or several of the autoantigens -GAD65, IA-2, IAAs, ICA or ZnT8 signals an autoimmune pathogenesis of β-cell destruction and indicates condition‘s severity, the presence of multiple autoantibodies having the highest positive predictive value for type 1 diabetes [6, 21, 22].

The presence of GAD65 in type 1 diabetes was shown to depend on age at diagnosis and gender: GAD65 positive female diabetic patients have higher GAD65 levels and a more severe loss of β-cell function than male patients with the same age at diagnosis [23]. GAD65 is less frequent among boys developing diabetes before the age of 10 years, with diagnostic sensitivity over 80% in both males and females in older children, teenagers, and young adults [22]. In our cohort, highest frequency of positive GAD65 antibodies was in patients with disease onset between 14 and 19 years (66.4%).

We have found positive GAD65 antibodies in 52.7%, IA-2 in 57.8%, IAAs in 73.9%, and ICAs in 7% of cases; 36.5% of patients had both GAD65 and IA-2 antibodies. *Pardini* et al have reported positive GAD65 in 80.0%, IA-2 - in 62.9% and both GAD65 and IA-2 - in 82.9% of cases with recent-onset type 1 diabetes. The frequency of positive antibodies was lower in cases with long duration of type 1 diabetes [24]*,*which is similar to our findings: all types of antibodies had tendency to be lower in subjects with longer duration of diabetes, and highest proportion of all negative antibodies was found in patients with longest duration of type 1 diabetes.

It has previously been reported that frequency of positive ICAs declines following diagnosis, and remains positive in less than 5–10% of type 1 diabetes patients after 10 years [25]*.* On the contrary, in our study frequency of positive ICAs was surprisingly low in the 0–4 years disease duration group (5.4%), and much higher in those with 15–19 years duration (11.1%). Several longitudinal studies indicate that type 1 diabetes patients remain GAD65 and/or IA-2 positive up to 80% after 12 years [26, 27].

Ketosis at the time of diagnosis is a typical feature of type 1 diabetes, and was present in 87.3% of cases in our study. It was less frequent among youth with family history of diabetes, which is probably due to alertness of family members to initial signs and symptoms of the disease. Also, ketosis was less frequent finding at diagnosis in patients with non-autoimmune diabetes, which might indicate a different cause and mechanism of hyperglycemia, at least in part of these patients group.

Most cases of type 1 diabetes occur sporadically in the absence of family history of diabetes [28]. The empiric risk of being affected if a first-degree relative has diabetes is 5% [29]. Although more than 85% of patients with type 1 diabetes lack a positive family history, a high familial clustering with a mean prevalence of 6% in siblings is usually found [30]*.* In our study, the frequency of first-degree relatives with either types of diabetes was 10.8% in the whole cohort, and was significantly higher in patients with negative autoantibodies (24.1% vs. 9.4%, *p* < 0.001), possibly indicating different pathways of disease inheritance.

Nonproliferative retinopathy was found in 8.2% of our patients, and was observed in older patients with longer duration of diabetes. Although the facilities for the management of diabetes are constantly improving, the risk of retinopathy remains high. The Oulu cohort study reports extremely high prevalence of diabetic retinopathy and proliferative diabetic retinopathy (94% and 35%, respectively) in patients who have had diabetes since childhood after 18 years of follow up [31]. Interestingly, we found significantly higher frequency of all negative antibodies among patients with retinopathy in our cohort, indicating a similar or even higher risk for development of this complication in antibodies-negative forms of diabetes. We also found higher levels of IA-2 antibody and IAAs levels among patients without retinopathy, this could be related with higher residual β-cell function, as reported by other authors, although they mainly analyzed inverse relationship between GAD65 antibody levels and severe retinopathy in young type 1 diabetic patients [32].

Neuropathy was present in 8.8% of cases in our study. Autoimmunity status was not different in patients with and without neuropathy. Similar frequency of neuropathy (8.2%) was found in type 1 diabetes patients in SEARCH pilot study [33], although low reproducibility of vibration perception threshold values in young age was previously reported [34].

Elevated AER was found in 94 (8.1%) cases of our young cohort, and was linked to older age at study entry, longer disease duration, higher HbA1c and higher daily insulin dose. Presence of pancreatic autoantibodies was again not related to elevation of AER, indicating comparable risk of diabetic nephropathy in both antibodies-positive and antibodies-negative diabetes, which seems to be more related to diabetes duration and control. A more intensive follow-up is needed in these cases, since the data from Finnish FinnDiane study showed that almost all of the excess mortality seen in type 1 diabetes was related to the development of micro- or macroalbuminuria [35]. Similar results were reported in The Pittsburgh Complications Study [36]. On the other hand, frequency of increased AER in our study was twice lower in the antibodies-negative group, and possibly did not reach statistical significance because of small number of patients with elevated AER in this group.

We must admit that the main weakness of our study was measurement of pancreatic antibodies in both newly diagnosed patients and in patients who were already on insulin treatment, which was partly responsible for high frequency of IAAs in our cohort. Therefore, a proportion of subjects with positive only IAAs might in fact have non-autoimmune form of diabetes, as one patient with neonatal diabetes positive for IAA. Moreover, even in presence of autoimmune markers, the likelihood of monogenic diabetes origin cannot be excluded, as demonstrated in some studies [37]. On the other hand, ZnT8 antibodies have not been determined in our cohort, implying that some subjects with negative antibodies might include individuals with undetected autoimmunity. Furthermore, reduction in levels and even disappearance of autoantibodies in the course of the disease is a well known phenomenon in diabetic patients, making the subject even more complex.

Another study limitation is that according to national type 1 diabetes register the study population covered 70% of young 18–25 years old adults, since at this age many young individuals are moving abroad or are busy in their work and therefore are not available for investigations. Nevertheless, we screened all pediatric and 70% of young adult population with type 1 diabetes in Lithuania, identifying their autoimmune status, metabolic control and diabetes complications. This extensive characterization of the cohort will allow us to proceed to genetic testing of subjects with antibodies-negative diabetes for eventual identification of monogenic forms, which might enable an optimization of treatment and further follow-up of such patients.

## Conclusions

In summary, no immunological markers of beta-cell autoimmunity were found in 7.5% of our cohort of young T1D patients, and in 12.2% of newly diagnosed diabetic patients. Antibodies-negative patients with presumed type 1 diabetes in this study had higher incidence of family history of diabetes, higher frequency of retinopathy, less frequent ketosis at presentation, but similar age at onset, HbA1c, incidence of nephropathy and neuropathy compared to antibodies-positive patients. Further genetic testing in antibodies-negative patients needs to be performed in order to elucidate the origin of the disease. Nevertheless, optimal diabetes management remains the most important way to prevent development of microvascular complications and to achieve better quality of life in all patients with diabetes.

## Abbreviations

AER: Albumin excretion rate
ATPO: Thyroid peroxidase antibodies
GAD65: Antibodies against glutamic acid decarboxylase antigen 65 kD
IA-2: Antibodies against protein tyrosine phosphatase
IAAs: Insulin antibodies
ICAs: Islet cell antibodies
T1D: Type 1 diabetes
tTG-A: Tissue transglutaminase antibodies
ZnT8A: Zinc transporter 8 antibodies
